# Human amniotic epithelial cell feeder layers maintain iPS cell pluripotency by inhibiting endogenous DNA methyltransferase 1

**DOI:** 10.3892/etm.2013.1279

**Published:** 2013-09-02

**Authors:** QING CHEN, CHAOLIN QIU, YONGYI HUANG, LIZHEN JIANG, QIN HUANG, LIHE GUO, TE LIU

**Affiliations:** 1 Shanghai Pudong New Area Gongli Hospital, Shanghai 200135;; 2 Institute of Biochemistry and Cell Biology, Shanghai Institute for Biological Sciences, Chinese Academy of Sciences, Shanghai 200031;; 3 Sino-America United Stem Cell Research Center, Shanghai 200333;; 4 Shanghai Geriatric Institute of Chinese Medicine, Longhua Hospital, Shanghai University of Traditional Chinese Medicine, Shanghai 200031, P.R. China

**Keywords:** human amniotic epithelial cells, human induced pluripotent stem cells, DNA methyltransferase 1, pluripotency maintenance

## Abstract

Maintaining induced pluripotent stem (iPS) cells in an undifferentiated, self-renewing state during long-term cultivation is, at present, a major challenge. We previously showed that human amniotic epithelial cells (HuAECs) were able to provide a good source of feeder cells for mouse and human embryonic or spermatogonial stem cells; however, the epigenetic mechanisms have not been elucidated. In the present study, mouse embryonic fibroblasts (MEFs) and HuAECs were compared as feeder layers for the long-term culture of human iPS cells. The HuAEC feeders allowed human iPS cells to maintain a high level of alkaline phosphatase (AP) activity and to express key stem cell markers during long-term subculture whereas the MEF feeders did not,. Moreover, the HuAEC feeders significantly affected the cell cycle regulation of the iPS cells, maintaining them in the resting stage and the early stage of DNA synthesis (G0/G1 stage). Furthermore, the CpG islands of the *Nanog* and *Oct4* promoters were hypomethylated, while the *Nanog*- and *Oct4*-specific loci exhibited higher levels of histone H3 acetylation and lower levels of H3K27 trimethylation in iPS cells cultured on HuAECs compared with those cultured on MEFs. The DNA methyltransferase 1 (DNMT1) expression in iPS cells cultured on HuAECs was shown to be lower than in those cultured on MEFs. In addition, DNMT1-silenced human iPS cells were able to maintain pluripotency over long-term culture on MEFs. In combination, these results suggest that endogenous DNMT1 expression in human iPS cells may be regulated by HuAEC feeder cells and that *Nanog* and *Oct4* are crucial components required for the maintenance of iPS cells in an undifferentiated, proliferative state, capable of self-renewal.

## Introduction

Induced pluripotent stem (iPS) cells, generated from murine somatic fibroblast cells by Takahashi and Yamanaka (1), have revolutionized modern science (1–9). The iPS cells represent an appealing option for the derivation of pluripotent patient-specific cells, as no clinically useful embryos or oocytes are employed, which thereby avoids ethical obstacles (10). The original iPS cells were generated by viral transduction of a limited set of transcription factors (Oct3/4, Sox2, c-Myc and Klf4; or Oct3/4, Sox2, Lin28 and *Nanog*), which reprogrammed somatic cells into pluripotent, embryonic stem cell (ESC)-like cells (10). Since then, iPS cells have been generated in different species by a number of methods (6,8,11–17), sharing with ESCs the key properties of unlimited self-renewal and pluripotency. Although human iPS cells may become a more readily available source of cells for clinical treatment, the question of whether they are able to maintain their cell self-renewal and pluripotency during *in vitro* culture remains. In our previous studies, we indicated that the expression of numerous growth factors, including basic fibroblast growth factor (bFGF), epidermal growth factor (EGF) and insulin-like growth factor 1 (IGF-1), and leukemia inhibitory factor (LIF) by human amniotic epithelial cells (HuAECs) may be crucial for the function of feeder cells in maintaining mouse and human ESCs, as well as mouse spermatogonial stem cells, in an undifferentiated, proliferative state, capable of self-renewal (18–21). Furthermore, we have demonstrated that HuAEC-dependent epigenetic modifications of the *c-Myc* gene locus occur in the previously mentioned stem cells, providing a possible mechanism for their HuAEC-dependent maintenance in an undifferentiated state (18–20). Although we previously demonstrated that HuAECs were able to be effectively used as feeder cells, very little is known about how they maintain iPS cell self-renewal and inhibit the differentiation of the iPS cells.

In a previous study, *Nanog* and *Oct4* were shown to be two key factors required to maintain the pluripotency of ESCs, iPS cells and early embryos; they are co-expressed in developmental stage- and cell type-specific manners (22). The *Nanog* gene is expressed in pluripotent cells, including ESCs, embryonic carcinoma and embryonic germ cells, and its transcripts are present in the interior cells of the compacted morula and the inner cell mass of the blastocyst (22). *Oct4* is also necessary for maintaining the pluripotency of cells of inner cell mass lineage (22), and its expression has also been observed in ESCs and iPS cells. The reduction in *Oct4* expression leads to trans-differentiation of ESCs into trophoblast stem cells under adequate culture conditions (22). Previous studies have proposed that partial DNA demethylation in restricted areas in the *Oct4* regulatory region is required for gene activation (3,23–26). The *Nanog* promoter is also demethylated in nuclear transfer ESCs, fibroblast ESCs and in transduced cells (3,23,27). Moreover, DNA methyltransferase (DNMT)-1 and DNMT3 (a/b) have been shown to contribute synergistically to the methylation of *Oct4* and *Nanog* during mouse embryonic cell differentiation *in vivo* (28).

Epigenetic regulation, particularly DNA methylation, is crucial in gene silencing in mammals (28). DNA methylation is important for establishing the dynamic chromatin configuration of the genome in pluripotent ESCs and iPS cells and for coordinating genomic reorganization during cell differentiation (29). A number of key proteins have been shown to affect epigenetic modifications via DNA methylation, most importantly the DNA methyltransferases, DNMT1, DNMT3a and DNMT3b (30). DNMT1 is the ‘maintenance methyltransferase’ that localizes to replication foci during the S phase and copies the DNA methylation pattern to the newly synthesized daughter strand (31,32). DNMT3a and DNMT3b are *de novo* methyltransferases, responsible for the methylation of unmodified DNA (31,32). Sen *et al* (33) have indicated that the DNMT1 protein is predominantly confined to cells of the basal layer of adult human epidermal tissue and is absent from the outer differentiated layer. Therefore, DNMT1 is expressed in epidermal progenitor-containing cell populations and is lost during differentiation (33). However, a DNMT1, DNMT3a and DNMT3b triple-knockout ESC line was shown to grow robustly and maintain its undifferentiated characteristics (29). In addition, when ESCs or iPS cells are treated with 5-aza-cytidine (a DNA methyltransferase inhibitor), the influence of DNMT1 is weakened and DNA hypomethylation occurs during cell reprogramming (34). Although DNMT1 is frequently designated as a maintenance methyltransferase, while DNMT3a and DNMT3b are classified as *de novo* methyltransferases, these enzymes have been shown to exhibit overlapping functions (29). Moreover, in spite of a 5-to-30-fold higher preference of DNMT1 for hemimethylated DNA, it exhibits greater *de novo* DNA methyltransferase activity *in vitro* and is present at higher levels than DNMT3a and DNMT3b in ESCs and somatic cells (35).

Experimentally, human iPS cells are highly similar to human ESCs in terms of morphology, proliferation, gene expression and the epigenetic status of pluripotency-specific genes (21). Furthermore, the global epigenetic landscapes, as indicated by the distribution of histone modifications and DNA methylation, are very similar between ESCs and iPS cells (29). Therefore, the cells employ the same molecular mechanisms to maintain the expression of the pluripotency regulators *Nanog* and *Oct4* and to maintain their properties via epigenetic modifications (36). Our preliminary experiments revealed that HuAECs were able to be effectively used as feeder cells to maintain iPS cell self-renewal and inhibit iPS cell differentiation. iPS cells simultaneously express high levels of *Oct4* and *Nanog* when cultured on HuAECs. Accordingly, we hypothesized that the low endogenous activity of DNMT1, DNMT3a and/or DNMT3b in human iPS cells may lead to hypomethylation of the CpG islands on the promoter regions of *Nanog* and *Oct4* and that the high expression of these factors, modulated by HuAECs feeder layers, may maintain the pluripotency and self-renewal properties of the iPS cells.

## Materials and methods

### Preparation of mouse embryonic fibroblasts (MEFs) and HuAECs

MEF cells were isolated from 13-day-old C57BL/6 mouse embryos. Cells were mitotically inactivated using mitomycin C (Sigma-Aldrich, St. Louis, MO, USA), as described previously (18). The mitotic inactivation of MEF cells was conducted by treatment with 10 *μ*g/ml mitomycin C for 2 h at 37°C. The cells were washed three times with phosphate-buffered saline (PBS), digested with 0.25% trypsin-EDTA solution (cat no. 25300-054; Invitrogen Life Technologies, Carlsbad, CA, USA) and plated at a density of 1×10^5^/ml, with 2.5 ml in each well of a gelatin-coated six-well dish. Human placentas were obtained with written and informed consent from pregnant females who were negative for human immunodeficiency virus (HIV)-I, hepatitis B and hepatitis C. The study received approval for the appropriate use of human amnion by the institutional Ethics Committee of Shanghai Geriatric Institute of Chinese Medicine (Shanghai, China). Amniotic membranes were mechanically separated from the chorions of placentas, which were obtained from females who had undergone an uncomplicated Cesarean section. HuAECs were harvested from the epithelial layers (with the basement membrane attached) of the obtained amniotic membranes, as described in a previous study, with certain modifications (18). In brief, the membrane was placed in a 250-ml flask containing Dulbecco’s modified Eagle’s medium (DMEM) and cut with a razor to produce 0.5–1.0 cm^2^ segments. The segments were subsequently digested with 0.25% trypsin-EDTA at 37°C for 45 min and the resulting cell suspensions were seeded in a six-well plate in DMEM supplemented with 10% fetal calf serum (FCS; PAA Laboratories GmbH, Pasching, Austria), penicillin (100 U/ml) and glutamine (0.3 mg/ml). Following this, the cells were incubated in a humidified tissue culture incubator containing 5% CO_2_ at 37°C. The HuAECs were grown to a density of ~100% and were subsequently used as feeder layers for human iPS cell culture following mitomycin C (Sigma-Aldrich) treatment.

### Co-culture of human iPS cells with HuAECs and MEFs

The human iPS cells were generated by our laboratory and derived from CD34^+^ human amniotic fluid cells (HuAFCs) via transduction with lentiviral constructs encoding only *Oct4*, as previously described (37). iPS cultures were separated from the feeder cells by treatment with 0.125% trypsin-EDTA solution and plated onto and co-cultured with HuAECs or MEFs. The cells were cultured in DMEM:F12 (1:1) medium supplemented with 15% KnockOut™ Serum Replacement (Invitrogen Life Technologies), 1 mM sodium pyruvate, 2 mM L-glutamine, 0.1 mM nonessential amino acids, 0.1 mM β-mercaptoethanol, penicillin (25 U/ml)-streptomycin (925 mg/ml), and mixed, without LIF. The cells were incubated in a humidified tissue culture incubator containing 5% CO_2_ at 37°C. All cells were cultured on the same feeder until the 10th passage, prior to being used for subsequent experiments.

### Alkaline phosphatase (AP) staining

The AP activity of human iPS cells, which were cultured on HuAECs or MEFs, was determined using an alkaline phosphatase detection kit (Sigma-Aldrich), in accordance with the manufacturer’s instructions (38).

### RNA extraction and analysis using quantitative polymerase chain reaction (qPCR)

Total-RNA from each cell was isolated using TRIzol reagent^®^ (Invitrogen Life Technologies), in accordance with the manufacturer’s instructions. The RNA samples were treated with DNase I (Sigma-Aldrich), quantified and reverse-transcribed into complementary DNA (cDNA) with the ReverTra Ace-α First Strand cDNA Synthesis kit [Toyobo (Shanghai) Biotech Co., Ltd., Shanghai, China]. qPCR was conducted with a RealPlex4 real-time PCR detection system from Eppendorf (Hamburg, Germany), with SYBR^®^ Green Real-Time PCR Master Mix [Toyobo (Shanghai) Biotech Co., Ltd.] as the detection dye. The qPCR amplification was performed over 40 cycles with denaturation at 95°C for 15 sec and annealing at 58°C for 45 sec. The target cDNA was quantified using the relative quantification method. A comparative threshold cycle (Ct) was used to determine gene expression relative to a control (calibrator); steady-state mRNA levels are expressed as an n-fold difference relative to the calibrator. For each sample, the maker gene Ct values were normalized with the formula: ΔCt=Ct_genes - Ct_18S RNA. To evaluate the relative expression levels, the following formula was used: ΔΔCt=ΔCt_all_groups - ΔCt_blank_control_group. The values used to the plot relative expression of the markers were calculated using the expression 2^−ΔΔCt^ method. The mRNA levels were calibrated on the basis of levels of 18S ribosomal RNA (rRNA). The cDNA of each gene was amplified with primers as previously described (21,37,39,40).

### RNA interference and transfection

The small interfering RNA (siRNA)-DNMT1 plasmid was manufactured by Shanghai GenePharma, Ltd. (Shanghai, China) and the methods used for plasmid transfection were in accordance with the company’s instructions. In brief, iPS cells were cotransfected with 0.3 *μ*g siRNA-DNMT1 expression plasmid or siRNA-Mock plasmid, respectively, using Lipofectamine 2000 reagent (Invitrogen, Life Technologies Corporation, Grand Island, NY, USA), in accordance with the manufacturer’s instructions. The cells were seeded in a six-well plate and cultured in DMEM:F12 (1:1) medium supplemented with 15% KnockOut™ Serum Replacement, 1 mM sodium pyruvate, 2 mM L-glutamine, 0.1 mM nonessential amino acids, 0.1 mM β-mercaptoethanol, penicillin (25 U/ml)-streptomycin (925 mg/ml) and mixed, without LIF. The cells were incubated in a humidified tissue culture incubator containing 5% CO_2_ at 37°C until 80% confluence was achieved.

### Flow cytometric (FCM) analysis of cell cycle by propidium iodide (PI) staining

Each group of cells was seeded at a density of 3×10^5^ cells per well in six-well plates and cultured until 85% confluent. Following this, each group of cells was washed three times with PBS, prior to being subjected to centrifugation (Allegra X-22^®^; Beckman Coulter, Miami, FL, USA) at 1,000 × g for 5 min. The cell pellets were then resuspended in 1 ml PBS, fixed in 70% ice-cold ethanol and stored in a freezer for >48 h. Prior to FCM analysis, the fixed cells were centrifuged, washed twice with PBS and resuspended in PI staining solution (Sigma-Aldrich) containing 50 *μ*l/ml PI and 250 *μ*g/ml RNase A (Sigma-Aldrich). The cell suspensions, which were hidden from light, were incubated for 30 min at 4°C and analyzed using a fluorescence-activated cell sorter (FACS; FCM-500; Beckman Coulter). A total of 20,000 events were acquired for analysis using CellQuest software (BD Biosciences, Franklin Lakes, NJ, USA).

### Bisulfite conversion of genomic DNA and methylation-specific PCR (MS-PCR)

The cells were lysed in DNA lysis buffer [0.5% sodium dodecyl sulfate (SDS), 0.1 M EDTA, 10 mM Tris-HCl (pH 8.0) and 100 ng/ml proteinase K; all from Sigma-Aldrich] and incubated at 55°C for 2 h. The treatment of genomic DNA and the MS-PCR assay were performed as previously described (2,5,9,18,37). In addition, the specific primers for *Nanog* and *Oct-4* were designed as previously described (2,5,9,18,37). The PCR products were separated using 12 g/l ethidium bromide containing agarose gel electrophoresis with 1X Tris acetate EDTA (TAE) buffer, and visualized under UV illumination.

### Chromatin immunoprecipitation (ChIP) assays

ChIP experiments were conducted using anti-acetylated histone H3 antibody (Upstate Biotechnology, Inc., Lake Placid, NY, USA), anti-trimethylated H3K27 antibody (Abcam, Cambridge, UK) and normal rabbit immunoglobulin G (IgG; Upstate Biotechnology, Inc.) as a negative control. All steps were performed as previously described (2,5,9,18,37). The cells were fixed using 1% formaldehyde for 30 min at 37°C and then quenched using 125 mM glycine for 10 min at room temperature to form DNA-protein cross-links. Following this, the samples were placed on ice and sonicated until chromatin fragments became 200–1,000 bp in size. The samples were then incubated with antibodies at 4°C overnight. The PCR amplification was performed under the following conditions: 33 cycles of denaturation at 95°C for 30 sec, annealing at 55°C for 30 sec and extension at 72°C for 30 sec.

### Immunofluorescence (IF) staining

The cultured cells were washed three times with FCS and fixed with 4% paraformaldehyde (Sigma-Aldrich) for 30 min. The cells were then washed using Tris-buffered saline containing 0.1% Triton X-100 [TBST-100 buffer; 25 mM Tris-HCl (pH 8.0), 125 mM NaCl and 0.1% Triton X-100] three times, prior to blocking. Following blocking, the cells were incubated with rabbit anti-human Oct3/4 polyclonal antibody (1:200; Chemicon, Temecula, CA, USA) and rabbit anti-human *Nanog* polyclonal antibody (1:200; Chemicon) overnight at 4°C, and then with Cy3-conjugated goat anti-rabbit IgG antibody (1:200; Abcam) and 5 *μ*g/ml 4’,6-diamidino-2-phenylindole (DAPI; Sigma-Aldrich) at room temperature for 30 min. Following this, the cells were thoroughly washed with TBST-100 and viewed under a fluorescence microscope (DMI3000; Leica Camera Inc., Allendale, NJ, USA).

### Western blot analysis

Cells were lysed using a 2X loading lysis buffer [50 mM Tris-HCl (pH 6.8), 2% sodium dodecyl sulfate, 10% β-mercaptoethanol, 10% glycerol and 0.002% bromphenol blue]. The total quantity of proteins from the cultured cells was subjected to 12% SDS-polyacrylamide gel electrophoresis (PAGE) and transferred onto Hybrid-polyvinylidene fluoride (PVDF) membranes (Millipore, Bedford, MA, USA). Following blocking with 5% (w/v) non-fat dried milk in Tris-buffered saline containing Tween-20 [TBST-20; 25 mM Tris-HCl (pH 8.0), 125 mM NaCl and 0.05% Tween-20], the PVDF membranes were washed four times (15 min each) with TBST-20 at room temperature and incubated with primary antibody. Following extensive washing, the membranes were incubated with horseradish peroxidase (HRP)-conjugated goat anti-rabbit IgG secondary antibody (1:1,000; Santa Cruz Biotechnology, Inc., Santa Cruz, CA, USA) for 1 h. The membranes were then washed four times (15 min each) with TBST-20 at room temperature, prior to the immunoreactivity being visualized by enhanced chemiluminescence (ECL) using an ECL Chemiluminescent Substrate Reagent kit from Perkin-Elmer Life Science (Norwalk, CT, USA).

### Teratoma formation

All animal procedures were conducted at Shanghai University of Traditional Chinese Medicine (Shanghai, China) with approval from the Institutional Animal Care and Use Committee and in accordance with the institutional guidelines. Human iPS cells (1×10^6^) were inoculated into the hind legs of severe combined immunodeficient (SCID) mice. Teratomas were embedded in paraffin and histologically examined following hematoxylin and eosin staining. The procedure for the teratoma formation experiment was performed as described previously (18).

### Statistical analysis

Each experiment was performed as least three times and the data are presented as the mean ± standard error (SE). The differences were evaluated using Student’s t-tests. P<0.05 was considered to indicate a statistically significant difference.

## Results

### Pluripotency of iPS cells derived from CD34^+^ HuAFCs

**Figure 1.**
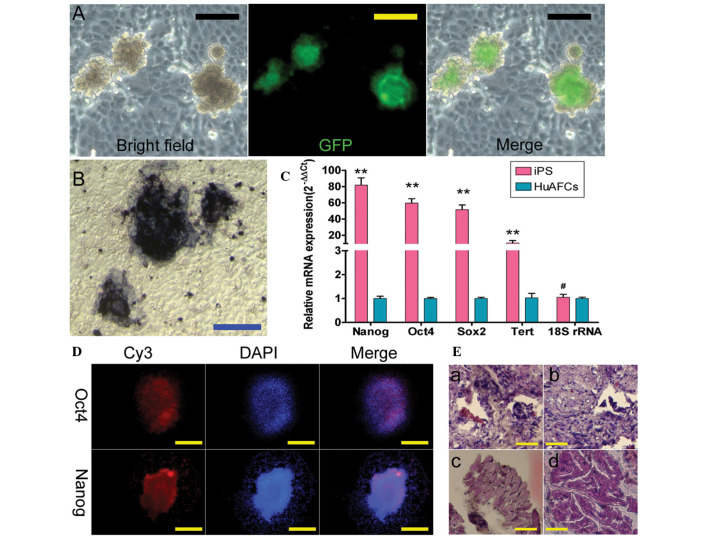
Characterization of reprogrammed human induced pluripotent stem (iPS) cells. (A) Bright-field image, green fluorescent protein (GFP)-positive clones and merged GFP and bright-field images of human iPS cells in the seventh phase of culture on human amniotic epithelial cell (HuAEC) feeder layers. Scale bar=50 *μ*m. (B) Deep blue staining for alkaline phosphatase (AP) activity in a human iPS cell colony. The AP activity in human iPS cells cultured on HuAECs was steady in the seventh phase. Scale bar=50 *μ*m. (C) Quantitative reverse transcription-polymerase chain reaction analysis of transcriptional expression of endogenous stem cell markers (*Oct4*, *Nanog*, Sox2 and Tert) in human iPS cells relative to human amniotic fluid cells (HuAFCs). ^**^P< 0.01 vs. HuAFCs; ^#^P>0.05 vs. HuAFCs; n=3. (D) Immunostaining was performed on human iPS cells using antibodies against *Nanog* and *Oct4*. Scale bar=50 *μ*m. DAPI, 4’,6-diamidino-2-phenylindole. (E) Histology of teratomas in severe combined immunodeficient mice and histology of a teratoma composed of ectodermal, endodermal and mesodermal tissue. Scale bar=100 *μ*m. (a and b) Adipose tissue (mesodermal) and medullary tube (ectodermal), respectively; (c) striated muscle (mesodermal); and (d) gastrula (endodermal).

We have previously described the successful generation of iPS cells from CD34^+^ HuAFCs by transduction with lentiviral constructs encoding only *Oct4* (37). In this study, the iPS cells were cultured on HuAEC feeder layers until the 10th passage, prior to use in experiments. After testing the effects of different feeder layers, the pluripotency of iPS cells was assayed. Under the microscope, a number of ESC-like colonies were observed among the feeder cells; these *Oct4*-green fluorescent protein (GFP)-positive colonies appeared isolated and rounded, consistent with more undifferentiated cells (Fig. 1A). Furthermore, the AP activity of these human iPS cells was high, as represented by the deep blue staining on the surface of the colonies (Fig. 1B). In addition, the IF staining revealed that the expression levels of the pluripotent stem cell markers, *Nanog*, *Oct4* and Sox2, were increased in the iPS colonies (Fig. 1D). Consistent with the IF results, qPCR analysis showed that the expression levels of these stem cell markers were ~60–100-fold higher in human iPS cells than in HuAFCs, which served as an internal control (Fig. 1C). Moreover, the high level of telomerase activity in human iPS cells suggested that their replicative life-span was likely to exceed that of somatic cells. Meanwhile, in the *in vivo* xenograft experiments, teratomas formed on the legs of SCID mice injected with the iPS cells (Fig. 1E). The iPS-derived teratomas contained cellular representatives of all three germ layers (Fig. 1E). Based on these results, it was concluded that the human iPS cells derived from HuAFCs possessed strong pluripotency.

### Pluripotency of iPS cells cultured on MEFs is weakened during successive subcultures in vitro

**Figure 2.**
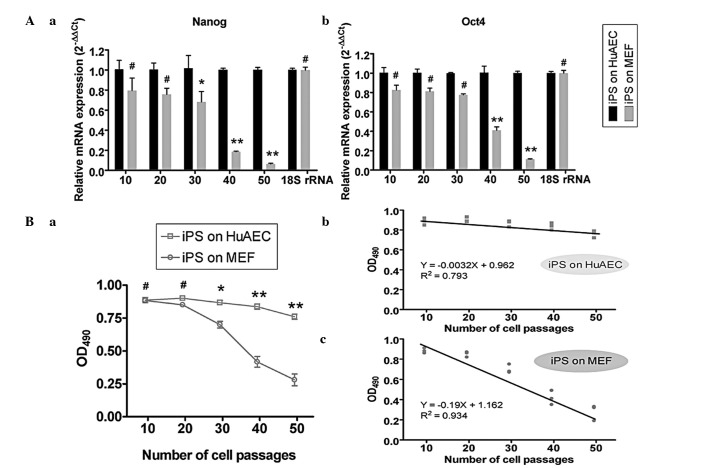
Expression of *Nanog* and *Oct4* and alkaline phosphatase (AP) activity in human induced pluripotent stem (iPS) cells at different passages. (A) Quantitative reverse transcription-polymerase chain reaction analysis showed that the levels of (a) *Nanog* and (b) *Oct4* expressed in the iPS cells cultured on mouse embryonic fibroblasts (MEFs) reduced rapidly, while those of iPS cells cultured on human amniotic epithelial cells (HuAECs) did not significantly change. Relative mRNA expression is shown following normalization to 18S ribosomal RNA (rRNA), serving as an internal control. ^*^P<0.05 vs. iPS cells on HuAECs; ^**^P<0.01 vs. iPS cells on HuAECs; ^#^P>0.05 vs. iPS cells on HuAECs; n=3. (Ba) AP activity assay of human iPS cells cultured on HuAECs or MEFs. The AP activity of human iPS cells cultured on MEFs (c) was decreased compared with those cultured on HuAECs (b) from the 30th to 50th passage; ^*^P<0.05 vs. iPS cells on MEFs; ^**^P<0.01 vs. iPS cells on MEFs; ^#^P>0.05 vs. iPS cells on MEFs; n=3. OD, optical density.

iPS cells were successively subcultured either on HuAECs or MEFs in order to evaluate the effect of the different feeder layers on pluripotency. The two groups of cells were cultured in uniform conditions and were subcultured in succession until passage 50. Since AP levels decrease as stem cells lose their pluripotency and differentiate, the AP activity of human iPS cells cultured on different feeder layers was analyzed at every 10th passage. The results showed that the AP activity of the iPS cells cultured on MEFs reduced rapidly and there was a significant negative correlation between the AP activity level and the number of cell passages (R^2^=0.934, P<0.05). However, when the iPS cells were cultured on HuAECs, their AP activity level was not significantly altered (R^2^=0.793, P>0.05; Fig. 2B). In addition, at the 40th and 50th passage, the AP activity levels of human iPS cells cultured on MEFs (0.417±0.041 and 0.280±0.045, respectively) were significantly lower than those on HuAECs (0.837±0.021 and 0.760±0.022, respectively). To evaluate whether the pluripotency of iPS cells changed, stem cells markers were assayed using qPCR. The results of the qPCR analysis showed that the *Nanog* and *Oct4* expression levels in iPS cells cultured on MEFs reduced rapidly, while those in iPS cells cultured on HuAECs did not markedly change over time (Fig. 2A).

### HuAEC and MEF feeders maintain iPS cells in the resting stage and early stage of DNA synthesis (G0/G1 stage)

**Figure 3.**
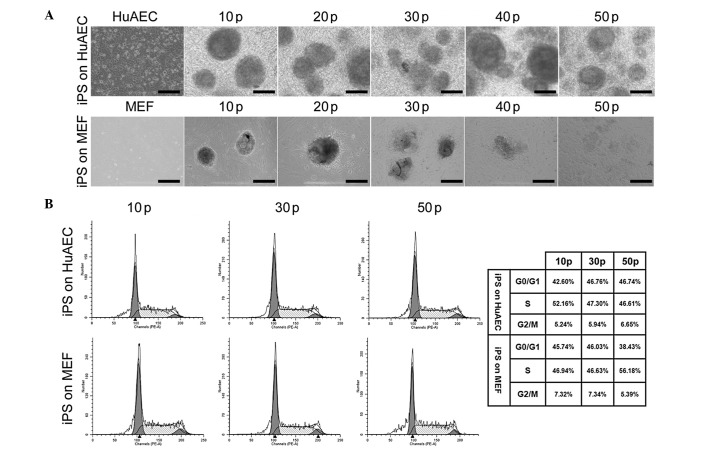
Morphology and cell cycles of human induced pluripotent stem (iPS) cells cultured on different feeder layers during subculture. (A) Morphology of iPS cells on human amniotic epithelial cells (HuAECs) or mouse embryonic fibroblasts (MEFs). Scale bar=50 *μ*m. On HuAECs, iPS colonies appeared more isolated and rounded with obvious boundaries with feeder cells, consistent with the appearance of undifferentiated cells during continuous culture. However, on MEFs, iPS colonies appeared to be migrating into the feeder layer with a less distinct cellular boundary, consistent with the appearance of more differentiated cells from the 40th passage. (B) The cell cycle of iPS cells cultured on MEFs or HuAECs was tested using flow cytometric analysis. The cell cycles of the iPS cells cultured on HuAECs were not markedly different between the 10th, 30th and 50th passages, indicating that long-term culture on HuAEC feeder layers did not affect the process of cell division in the iPS cells. Moreover, HuAEC feeder layers were able to maintain human iPS cells in the resting stage and early stage of DNA synthesis (G0/G1 stage). p, passage.

In the early passages (comparing passages 10, 30 and 50), the iPS cells grown on HuAECs and MEFs exhibited similar cell cycle distributions (Fig. 3). Furthermore, the cell cycles of the iPS cells cultured on HuAECs were not markedly different between the 10th, 30th and 50th passages, indicating that long-term culture on HuAECs feeder layers did not affect the process of cell division in the iPS cells. Similarly, when the iPS cells were cultured on MEFs, the FCM analysis showed no significant differences in the cell cycle distribution at each passage. The iPS cells cultured on MEFs were always in the resting stage and early stage of DNA synthesis (G0/G1 stage; Fig. 3). No significant differences were observed between the cell cycles of the iPS cells cultured on HuAECs or on MEFs at the 10th, 30th or 50th passages.

### Changes in DNA and histone epigenetic modifications in iPS cells during consecutive subcultures

**Figure 4.**
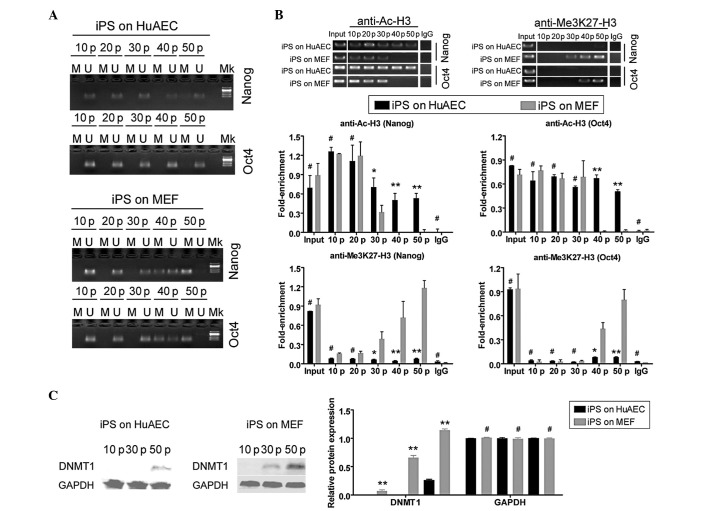
Epigenetic modifications of DNA and histones in human induced pluripotent stem (iPS) cells. (A) Analysis of the methylation state of the CpG islands of the *Nanog* and *Oct4* promoters using sodium bisulfite polymerase chain reaction (PCR) and sequencing. No significant differences in the DNA methylation status of the CpG islands of the *Nanog* and *Oct4* promoters were observed in human iPS cells cultured on human amniotic epithelial cells (HuAECs) compared with those cultured on mouse embryonic fibroblasts (MEFs) from the 10th to 30th passage. However, from the 40th passage, these regions were markedly hypermethylated in the iPS cells cultured on MEFs, although not on HuAECs. M, methylated CpG; U, unmethylated CpG; Mk, DNA marker. (B) Histone H3 acetylation and H3K27 trimethylation of the *Nanog* and *Oct4* loci. PCR-amplified genomic DNA obtained following immunoprecipitation with anti-acetylated histone H3 (anti-Ac-H3) antibody or anti-trimethylated histone H3K27 (anti-Me3K27-H3) antibody. ^*^P<0.05 vs. iPS cells on MEFs; ^**^P<0.01 vs. iPS cells on MEFs; ^#^P>0.05 vs. iPS cells on MEFs; n=3. (C) Western blot analysis showing that the expression of endogenous DNA methyltransferase 1 (DNMT1) in human iPS cells cultured on MEFs was significantly higher than that in iPS cells cultured on HuAECs following the 40th passage. ^**^P<0.01 vs. iPS cells on HuAECs; ^#^P>0.05 vs. iPS cells on HuAECs; n=3.p, passage; GAPDH, glyceraldehyde 3-phosphate dehydrogenase.

The DNA methylation status of the CpG islands in the *Nanog* and *Oct4* promoters was investigated using sodium bisulfite treatment of the iPS cells cultured on HuAECs or MEFs at every 10th passage. All the CpG islands in the *Nanog* and *Oct4* promoter regions were demethylated with no significant differences from the 10th to the 50th passage in human iPS cells cultured on HuAECs (Fig. 4A). However, in iPS cells cultured on MEFs, these regions of the *Nanog* and *Oct4* promoters were moderately demethylated at passages 10, 20 and 30 (Fig. 4A), prior to becoming hypermethylated at passages 40 and 50. These differences suggested that HuAECs positively regulated the *Nanog* and *Oct4* genes in human iPS cells by maintaining the hypomethylation of promoter CpG islands. In addition, ChIP assays were performed to evaluate the histone H3 acetylation levels of the *Nanog* and *Oct4* promoters in human iPS cells cultured on HuAECs or MEFs. In iPS cells cultured on HuAECs, the acetylation and K27 trimethylation of histone H3 in the *Nanog* and *Oct4* promoters and the 5’untranslated regions (5’UTRs) were similar to those in human iPS cells cultured on MEFs within 20 passages (Fig. 4B). However, from passage 30, histone H3 appeared relatively hyperacetylated and histone K27 appeared hypomethylated in the *Nanog*- and *Oct4*-specific sites in human iPS cells cultured on HuAECs, compared with the same regions in cells cultured on MEFs (Fig. 4B). These results suggested that HuAECs were capable of maintaining the *Nanog* and *Oct4* loci in an active transcriptional state through covalent histone modifications over long-term culture. Western blotting was also used to assess the relative protein expression of DNMT1 in iPS cells cultured on different feeder layers. Over multiple passages, DNMT1 protein was more highly expressed in iPS cells cultured on MEFs than in iPS cells cultured on HuAECs (Fig. 4C). At passages 30 and 50, the DNMT1 protein levels in iPS cells cultured on MEFs were 0.676±0.040 and 1.220±0.021 relative to glyceraldehyde 3-phosphate dehydrogenase (GAPDH) expression levels, respectively. These values were significantly higher than those in iPS cells cultured on HuAEC feeders (0.003±0.001 and 0.021±0.010 relative to GAPDH levels, respectively).

### Suppression of endogenous DNMT1 expression in iPS cells maintains their pluripotency while cultured on MEFs

**Figure 5.**
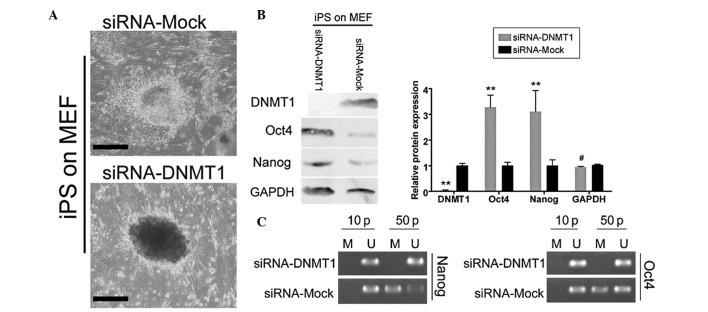
Suppression of endogenous DNA methyltransferase 1 (DNMT1) expression maintains the pluripotency of induced pluripotent stem (iPS) cells cultured on mouse embryonic fibroblast (MEF) feeder layers. (A) Morphology of small interfering RNA (siRNA)-DNMT1 or siRNA-Mock-transfected human iPS cells cultured on MEFs at the 50th passage (p). Scale bar=50 *μ*m. (B) Western blot analysis of the expression of endogenous *Oct4*, *Nanog* and DNMT1 in siRNA-DNMT1 or siRNA-Mock-transfected human iPS cells cultured on MEFs at the 50th passage. ^**^P<0.01, ^#^P>0.05 vs. siRNA-Mock-transfected cells; n=3. GAPDH, glyceraldehyde 3-phosphate dehydrogenase. (C) Analysis of the methylation state of the CpG islands of the *Nanog* and *Oct4* promoters using sodium bisulfite polymerase chain reaction and sequencing. M, methylated CpG; U, unmethylated CpG.

In order to evaluate whether endogenous DNMT1 expression promoted DNA methylation of *Nanog* and *Oct4* and the differentiation of cultured iPS cells, siRNA-DNMT1 and siRNA-Mock were transfected into iPS cells. The efficiency of the transfected siRNA on mRNA and protein expression was assessed using qPCR and western blotting, respectively, and these experiments were performed on all cell groups subcultured up to passage 50. As shown in Fig. 5B, western blotting indicated that the DNMT1 protein expression in iPS cells transfected with siRNA-Mock (1.002±0.089) was higher than that in iPS cells transfected with siRNA-DNMT1 (0.037±0.020, P<0.01, n=3). These results demonstrated that siRNA-DNMT1 specifically interfered with DNMT1 expression in iPS cells. Having confirmed that endogenous DNMT1 expression was suppressed by siRNA, the pluripotent stem cell biomarkers *Oct4* and *Nanog* in iPS cells cultured on MEFs were tested using western blotting. The results revealed that the *Oct4* and *Nanog* protein levels in the siRNA-DNMT1-transfected iPS cells cultured on MEFs were 3.277±0.475 and 3.108±0.719 of GAPDH expression levels, respectively (Fig. 5B). These values were significantly higher than those in the siRNA-Mock-transfected iPS cells (1.002±0.128 and 1.005±0.228 of GAPDH levels, respectively). In addition, the promoter regions of *Nanog* and *Oct4* were observed to be moderately hypomethylated or demethylated in siRNA-DNMT1-transfected iPS cells, while they were moderately hypermethylated in siRNA-Mock-transfected iPS cells (Fig. 5C). These differences suggested that DNMT1 positively regulated the *Nanog* and *Oct4* genes in human iPS cells through the *de novo* or maintained hypermethylation of the CpG islands, and that high DNMT1 expression in the iPS cells was able to promote their differentiation.

## Discussion

In the early stages of generation, iPS cells are generally highly efficient in their ability to be reprogrammed and possess a strong capacity for self-renewal. However, following consecutive subculturing, it was observed in this study that iPS cells began to lose these characteristics and to show decreased pluripotency as they differentiated, particularly following 40 passages. Moreover, when the iPS cells cultured on MEFs were subcultured continuously *in vitro*, the cells also showed a decreased expression of endogenous pluripotency stem cell markers, and the ability of the cells to differentiate into three germ layers *in vivo* was not as efficient as that of iPS cells cultured at earlier passages. Furthermore, assays at later passages indicated that the expression of two crucial transcriptor factors, *Oct4* and *Nanog*, decreased rapidly during the *in vitro* subculture of the iPS cells on MEFs. A previous study demonstrated that the promoter regions of *Oct4* and *Nanog*, as well as other pluripotency regulators, were methylated in somatic cells and became demethylated during reprogramming to a pluripotent state (29). The downregulation of *Nanog* and *Oct4* expression may induce the pluripotent stem cells to differentiate (41). The effects of DNA methylation on cells include transcriptional repression by the methylation of promoter regions, and this is required in mammals for embryonic development, X chromosome inactivation and imprinting (42). Moreover, a previous study revealed that three human DNA methyltransferases (DNMT1, DNMT3a and 3b) were widely expressed in a coordinated fashion in the majority of normal tissues, tumors and stem cells (42). In our previous studies, we have also demonstrated that the expression of a number of growth factors (bFGF, EGF and IGF-1) and LIF by HuAECs may be crucial components by which feeder cells maintain mouse and human ESCs, as well as mouse spermatogonial stem cells, in an undifferentiated, proliferative state, capable of self-renewal (18,21,37,39,40,43). With reference to these studies, we hypothesized that HuAECs as feeder layers may support the undifferentiated growth and maintain the pluripotency of iPS cells in long-term culture. The two main conclusions from this study are discussed in the following section.

DNMT1 was shown to induce hypermethylation of the promoters of *Oct4* and *Nanog*, although not those of *DNMT3a* or *3b*, causing the iPS cells to lose pluripotency during continuous subculture. We analyzed the expression levels of DNMT1 in iPS cells at different passages. Over time, the DNMT1 expression in these iPS cells became increasingly higher. Having confirmed that our siRNA specifically reduced endogenous DNMT1 expression in iPS cells, it was observed that the expression of pluripotent stem cell markers (*Nanog* and *Oct4*), as well as the level of AP activity, was higher in iPS cells transfected with siRNA-DNMT1 than those in iPS cells transfected with siRNA-Mock. Importantly, compared with the siRNA-Mock-transfected group, specific loci of *Nanog* and *Oct4* were demethylated/hypomethylated in the iPS cells transfected with siRNA-DNMT1 during long-term subculture. Therefore, we concluded that DNMT1, not DNMT 3a/3b, induced the hypermethylation of *Nanog* and *Oct4* and caused the iPS cells to lose pluripotency during long-term subculture.

A further conclusion from this study was that the use of HuAECs as feeder layers was able to maintain the pluripotency of human iPS cells during long-term subculture. It was demonstrated that the HuAEC feeder cells allowed human iPS cells to maintain a high level of AP activity. In addition, it was observed that expression levels of *Nanog*, *Oct4* and other important stem cell markers were higher in iPS cells cultured on HuAECs compared with those cultured on MEFs during long-term subculture. Furthermore, using the sodium bisulfite and PCR assay, it was demonstrated that the CpG islands of the *Nanog*- and *Oct4*-specific loci were hypomethylated in iPS cells cultured on HuAECs. In addition, iPS cells cultured on HuAECs exhibited higher levels of histone H3 acetylation and lower levels of H3K27 trimethylation at the *Nanog*- and *Oct4*-specific loci than those cultured on MEFs during subculture. Accordingly, the expression of DNMT1 in iPS cells cultured on HuAECs was lower than that in the cells cultured on MEFs. In combination, these results suggested that the HuAEC-induced epigenetic modifications at the *Nanog* and *Oct4* loci may be a key mechanism to maintain the iPS cells in an undifferentiated, proliferative state, capable of self-renewal, through the suppression of DNMT1 expression during long-term subculture *in vitro*.

Importantly, the use of HuAECs, in contrast to MEFs, as feeder layers avoids contamination from heterogeneous proteins. To date, numerous studies have indicated that the *in vivo* proliferation and differentiation of human iPS cells is dependent on a specific microenvironment, including various cytokines, LIF and other unknown factors (3,21,37). In order to maintain the self-renewal and proliferative properties and to inhibit the differentiation of iPS cells *in vitro*, a similar micro-environment must be provided with the essential ingredients for growth. HuAECs are temporary specialized fetal cells, derived from the placenta, which are able to maintain the pluripotency of early epiblast cells. Previous studies have indicated that HuAECs express a number of growth factors, such as LIF, EGF, bFGF, transforming growth factor (TGF)-α/β and bone morphogenetic protein (BMP)-4, as well as stem cell markers, including *Nanog*, *Oct4* and nestin (20,44). The results of our previous studies suggested that the expression of LIF by HuAECs was able to maintain mouse and human ESCs and mouse spermatogonial stem cells in an undifferentiated, proliferative state, capable of self-renewal (19,39,40). Additional advantages of the human placental amnion include its low toxicity and high safety, due to the presence of few exogenous foreign proteins. Furthermore, unlike ESCs, its use is free from ethical constraints. Therefore, this present study demonstrated that the human placental amnion may be used as an abundant source of feeder cells for human iPS cultures.
